# Effects of Glaucoma Stage on Optical Coherence Tomography Angiography Parameters in Patients With Primary Open-Angle Glaucoma

**DOI:** 10.7759/cureus.80583

**Published:** 2025-03-14

**Authors:** Burak Ozturk, Elif Erdem, Ibrahim Inan Harbiyeli, Ebru Esen, Selcuk Sizmaz, Begum Sulanc, Ozge Ozturk, Sevinc Puren Yucel Karakaya, Meltem Yagmur, Ayse Nihal Demircan

**Affiliations:** 1 Department of Emergency Medicine, Birmingham Heartlands Hospital, Birmingham, GBR; 2 Department of Eye Diseases, Faculty of Medicine, Çukurova University, Adana, TUR; 3 Department of Internal Medicine, Birmingham Heartlands Hospital, Birmingham, GBR; 4 Department of Biostatistics and Medical Informatics, Faculty of Medicine, Çukurova University, Adana, TUR

**Keywords:** macular vessel density, optical coherence tomography, optical coherence tomography angiography, peripapillary vessel density, primary open-angle glaucoma

## Abstract

Purpose

The purpose of this study is to evaluate the findings of optical coherence tomography angiography (OCTA) in patients with primary open-angle glaucoma (POAG) and investigate diagnostic power and correlations between the control group and patients at different stages of glaucoma.

Methods

This retrospective cross-sectional study included POAG patients and healthy volunteers as a control group. All subjects underwent a complete ophthalmological examination and computerised perimetry (GPc; Octopus 900, Haag-Streit AG, Köniz, Switzerland). Structural characteristics in the macular and peripapillary regions were assessed utilising optical coherence tomography (OCT; Cirrus HD-OCT 5000, Carl Zeiss Meditec, Dublin, CA, USA), and vascular parameters were assessed with OCTA (Optovue, RTVue XR100-2, Optovue Inc., Fremont, CA, USA). Additionally, the relationship between the glaucoma stage and OCTA findings was analyzed in patients and classified based on the global perimetry mean deviation value.

Results

The mean age of the POAG group (27 patients, 51 eyes) was 54.5±11.9 years, while the mean age was 45.1±9.7 years in the control group (54 patients, 54 eyes) (p=0.008). In terms of demographic features, the two groups did not differ significantly (p>0.05). The severity of the glaucoma was determined to be as follows: 24 eyes (47% of the total) were classified as early-stage, 12 eyes (23.5%) as moderate-stage, and 15 eyes (29.5%) as advanced-stage. OCTA radial peripapillary capillary (RPC) vessel density analyses showed that the inferior hemi's peripapillary vessel density (ppVD) area's under the receiver operating characteristic curve (AUC) (0.796 (0.665-0.927)) and the average ppVD (0.790 (0.658-0.921)) parameters had the best AUCs for early glaucoma detection. In the analysis of superficial macular vessel (SMV) density, all image upper-half (AI-UH), parafoveal upper-half (paf-UH), temporal (paf-T), superior (paf-S), and nasal (paf-N), as well as perifoveal upper-half (pef-UH), temporal (pef-T), and superior (pef-S) vessel densities were lower in moderate- and advanced-stage POAG eyes compared to healthy eyes (all p<0.05). Additionally, when glaucoma patients were analyzed together, SMV density measurements showed that AI, AI-UH, paf-UH, paf-T, paf-S, paf-N, pef, pef-UH, pef-T, and pef-N vessel densities were lower in advanced-stage POAG eyes compared to early-stage eyes (all p<0.05).

Conclusion

OCTA detected vascular damage in the macular and peripapillary regions in POAG patients. We found that ppVD helps identify early-stage glaucoma in RPC vessel density analysis. Moreover, macular vascular damage was observed to be more severe in advanced-stage glaucoma compared to early-stage glaucoma.

## Introduction

Primary open-angle glaucoma (POAG) is a chronic and progressive optic neuropathy, representing a significant cause of irreversible blindness globally [1]. POAG is characterised by retinal ganglion cell (RGC) and axon degeneration, which damages the optic nerve head (ONH) and causes vision field loss. Visual field testing and optical coherence tomography (OCT) are two examples of diagnostic procedures for glaucoma. These approaches concentrate on identifying functional deficiencies and structural alterations in the retinal nerve fibre layer (RNFL) and ganglion cell complex (GCC) of the eye [2]. While intraocular pressure (IOP) is a significant risk factor, vascular dysfunction has been recognised as an important contributor to the pathogenesis of POAG [3-6].

OCT angiography (OCTA), a non-invasive, repeatable, and reproducible imaging technique, has emerged as a promising tool for assessing retinal microvasculature. OCTA enables the quantification of vessel density in the macular and peripapillary regions, offering potential for earlier detection and monitoring of POAG [7-9]. Studies have demonstrated a reduction in peripapillary vessel density (ppPVD) and macular vessel density (MVD) in POAG patients, correlating with disease severity and visual field defects [6]. This suggests that vascular abnormalities detected by OCTA could provide information about disease progression, even before significant structural damage is apparent [6,7].

Given the importance of early diagnosis and consistent monitoring in preventing irreversible vision loss, this study intends to assess the vascular changes in the macular and peripapillary regions utilising OCTA across various stages of POAG. By comparing these findings with OCT measures, our study seeks to better understand the relationship between vascular and neural damage in glaucoma and assess the utility of OCTA as a complementary diagnostic tool [5,10].

## Materials and methods

Study groups

We performed a retrospective cross-sectional single-center study from February to June 2021 in the Ophthalmology Department at Çukurova University Hospital in Adana, Turkey. We included both healthy controls and patients with open-angle glaucoma between February and June 2021 in our study. Patient consent has been obtained both written and verbally between the above-mentioned time period. The principles outlined in the Declaration of Helsinki have been followed throughout the research process. Ethical approval was gained on 03/01/2025 with approval number 151. Refractive error between -3.00 and +3.00 diopters as well as the absence of corneal opacities, systemic disorders, cataract, intraocular surgery, history of trauma, and retinal pathology were considered as inclusion criteria for glaucoma and control groups. Eyes that have coexisting ophthalmologic conditions that cause the changed vision field were not included.

PAOG was diagnosed through distinctive alterations in the ONH identified via biomicroscopy and high-resolution OCT. This involved apparent changes in every aspect of the iridocorneal angle and pathological variances of the neuroretinal margin, severe papillary excavation, modified vascular paths, peripapillary atrophy, and defects in the RNFL adjacent to the optical disc margin. A normal anterior segment on the slit lamp and an open angle on gonioscopy were the other diagnostic criteria. The eyes of the control group had normal ONH examinations, IOP less than 21 mmHg, normal RNFL values, and a normal visual field.

The participants enrolled in this study went trough comprehensive ophthalmic inspection, which comprised visual acuity, refractive error, IOP measured with Goldmann applanation tonometer, anterior segment examination by slit lamp, fundus examination, ONH evaluation, and gonioscopy. Using the 30-2 threshold test, the automated standard visual field test was conducted with the support of a field analyzer (Octopus 900, Haag-Streit AG, Köniz, Switzerland). The division of glaucomatous eyes into three groups was based on the Hodapp-Parrish-Anderson rating scale of the severity of defects in visual field test: early mean deviation (MD) < 6 dB, moderate 6 dB < MD < 12 dB, and severe MD > 12 dB.

OCT and OCTA: data acquisition

In this study, structural features were evaluated with OCT (Cirrus HD-OCT 5000, Carl Zeiss Meditec, Dublin, CA, USA), and vascular parameters were evaluated with OCTA (Optovue, RTVue XR100-2, Optovue Inc., Fremont, CA, USA) in the macular and peripapillary regions. Circumpapillary RNFL and macular GCC thickness measurements were applied to all participants by using Cirrus HD-OCT 5000. We examined the average and four-sectoral thicknesses of the RNFL, which included superior, inferior, temporal, and nasal, as well as the average and sectoral thicknesses of the GCC, which included inferior and superior. Cubes measuring 4.5 mm by 4.5 mm were utilised for papillary OCTA. Three different locations were examined in order to determine the vascular density of the papillary region. A circle with a diameter of 1.5 millimetres was centred on the optic disc in the ONH layer. This indicates the inside disc vessel density (idVD). At the radial peripapillary capillary (RPC) layer, which has a diameter of 750 μm, both the average and sectoral ppVDs were assessed. These measurements were taken between two concentric circles with diameters of 1.5 mm and 2.25 mm, which were at the middle of an optic disc. In addition to this, we also performed an analysis of the vessel density in the RPC layer, which is referred to as the whole en face disc vessel density (wdVD). This analysis was performed on the full 4.5 mm by 4.5 mm disc scan picture.

The split-spectrum amplitude-decorrelation angiography (SSADA) algorithm captures the dynamic motion of moving particles, such as red blood cells flowing in a blood vessel, and allows high-resolution three-dimensional visualisation of perfused vasculature [11]. It also improves the signal-to-noise ratio (SNR) of flow detection [12]. For this reason, we used the SSADA algorithm as an image acquisition technique. Through the use of an automated thresholding system, binary pictures of vascular networks were generated.

To evaluate the macular region, 6x6 mm cubes were used in OCTA. The vascular density of the macular region was assessed in the superficial capillary plexus across three areas: the foveal vascular density (fVD) at the foveal region, the average and sectoral parafoveal vessel densities (pafVD), and the average and sectoral perifoveal vessel densities (pefVD). The entire 6x6 mm macula scan is referred to as the whole macula vessel density (wmVD).

Statistical analysis

Continuous variables are summarised using mean, standard deviation, median, and minimum-maximum. Categorical variables are represented as percentages and numbers. The Kolmogorov-Smirnov test was utilised to assess the normality of distributions for continuous variables. Depending on the fulfilment of statistical assumptions, comparisons among more than two groups were conducted using either one-way analysis of variance (ANOVA) or the Kruskal-Wallis test. For normally distributed data with homogeneity of variances involved, the Bonferroni and Games-Howell tests were used for group comparisons. For data that did not meet normality assumptions, the Mann-Whitney U test with Bonferroni adjustment was employed for multiple group comparisons.

To evaluate the diagnostic accuracy of OCTA vessel density parameters across the control and glaucoma groups (categorised as total, early, moderate, and severe stages), receiver operating characteristic (ROC) curve analysis was performed. The area under the ROC curve (AUC) was calculated along with a 95% confidence interval (CI). Correlations between different measurements were analyzed using Spearman’s rank correlation coefficient. All statistical analyses were conducted using IBM SPSS Statistics software (Version 20.0, IBM Corp., Armonk, NY, USA), and a p-value of 0.05 was established as the threshold for statistical significance.

## Results

Twenty-seven patients with glaucoma and 54 control patients were enrolled in the study. The mean age of the control and glaucoma patients were 45.1 ± 9.7 years (median: 46.0; min: 18.0; max: 63.0) and 58.2 ± 10.9 years (median: 59.0; min: 34.0; max: 81.0), respectively (p < 0.001). There were 26 males and 28 females in the control group and 15 males and 12 females in the glaucoma group (p = 0.694). Our research included 54 healthy eyes and 51 glaucoma eyes from 81 patients. Out of 51 glaucoma eyes, 24 were at the early stage, 45 at the moderate stage, and 15 at the severe stage. The mean duration of glaucoma was 8.8 years, with a range of 1 to 21 years. The RPC and en face macular (EM) vessel density parameters of the control and glaucoma groups are summarised in Tables 1-2, respectively. Table 1 shows that all papillary vessel density parameters of the patients in the control group were significantly higher than those of the patients in the early, moderate, and severe-stage glaucoma groups. In other words, lower RPC vessel density parameters were associated with more advanced glaucoma.

**Table 1:** Comparison of OCTA EM vessel density parameters between control and glaucoma groups. Data was expressed as median (min-max). ^a ^p<0.05 for A vs. B, ^b ^p<0.05 for A vs. C, ^c ^p<0.05 for A vs. D, ^d ^p<0.05 for B vs. D. wmVD: Whole en face macula vessel density; OCTA: Optical coherence tomography angiography; EM: En face macular; pafVD: Parafoveal vessel density; fVD: Foveal vessel density; pefVD: Perifoveal vessel density

EM vessel density (%)	Control group (A) (n=54)	Early glaucoma (B) (n=24)	Moderate glaucoma (C) (n=12)	Severe glaucoma (D) (n=15)	p-value
wmVD	48.85 (38.0-53.20)^a,b,c^	44.25 (35.50-52.90)^d^	37.60 (35.30-49.30)	35.45 (22.0-45.30)	<0.001
Superior hemi (wmVD)	48.55 (39.30-53.50)^b,c^	44.60 (37.90-52.20)^d^	38.55 (32.90-49.60)	37.05 (20.30-45.90)	<0.001
Inferior hemi (wmVD)	49.30 (36.90-53.20)^a,b,c^	44.55 (32.50-54.10)	39.15 (31.50-48.90)	35.25 (23.60-45.80)	<0.001
fVD	20.10 (1.1-35.60)^c^	18.50 (6.40-26.30)	22.20 (3.10-27.90)	14.65 (4.70-23.50)	0.018
pafVD average	51.70 (34.70-57.10)^a,b,c^	46.45 (35.90-56.10)	40.70 (34.20-51.30)	37.40 (23.50-48.50)	<0.001
Superior hemi (pafVD)	51.25 (35.40-56.30)^b,c^	46.75 (38.0-55.90)^d^	42.75 (36.10-51.70)	37.70 (25.0-48.20)	<0.001
Inferior hemi (pafVD)	51.90 (33.20-58.20)^a,b,c^	46.45 (33.70-57.30)	42.10 (32.20-50.90)	37.25 (21.90-50.0)	<0.001
Temporal (pafVD)	50.65 (33.80-56.90)^b,c^	46.95 (36.60-57.40)^d^	41.20 (34.50-50.70)	39.65 (26.20-48.90)	<0.001
Superior (pafVD)	52.0 (33.50-56.70)^b,c^	47.65 (36.50-55.50)^d^	44.05 (35.80-52.60)	37.45 (24.70-50.90)	<0.001
Nasal (pafVD)	50.85 (23.90-55.60)^a,b,c^	45.50 (36.60-57.70)^d^	41.40 (29.80-50.80)	36.80 (24.90-48.0)	<0.001
Inferior (pafVD)	52.55 (36.0-60.0)^a,b,c^	46.15 (28.90-57.30)	41.65 (29.80-51.0)	37.70 (16.80-48.70)	<0.001
pefVD	49.60 (39.0-54.10)^a,b,c^	45.0 (35.30-53.40)^d^	38.15 (35.0-49.90)	35.90 (2.20-46.60)	<0.001
Superior hemi (pefVD)	49.20 (39.60-54.0)^b,c^	45.25 (36.60-52.30)^d^	39.40 (33.70-50.0)	38.15 (20.10-46.20)	<0.001
Inferior hemi (pefVD)	50.20 (38.40-54.30)^a,b,c^	45.15 (33.40-54.80)	39.25 (32.0-49.0)	35.55 (24.20-46.90)	<0.001
Temporal (pefVD)	46.10 (31.80-50.60)^b,c^	41.80 (31.80-50.20)^d^	35.95 (27.80-44.80)	32.65 (23.70-44.30)	<0.001
Superior (pefVD)	49.0 (41.0-53.80)^b,c^	45.0 (37.30-53.70)^d^	37.15 (32.60-51.20)	37.30 (19.40-46.20)	<0.001
Nasal (pefVD)	53.99 (44.0-58.40)^a,b,c^	49.50 (37.30-56.30)^d^	43.60 (37.60-54.0)	42.35 (21.60-49.90)	<0.001
Inferior (pefVD)	50.0 (39.20-54.60)^a,b,c^	44.75 (30.70-55.80)^d^	38.90 (30.30-49.90)	32.90 (24.10-47.90)	<0.001

**Table 2:** Comparison of OCTA RPC vessel density parameters between control and glaucoma groups. Data is expressed as mean±standard deviation or median (min-max). ^a ^p<0.05 for A vs. B, ^b ^p<0.05 for A vs. C, ^c ^p<0.05 for A vs. D, ^d ^p<0.05 for B vs. D. wdVD: Whole en face disc vessel density; OCTA: Optical coherence tomography angiography; idVD: Inside disc vessel density; RPC: Radial peripapillary capillary; ppVD: Peripapillary vessel density

RPC vessel density (%)	Control group (A) (n=54)	Early glaucoma (B) (n=24)	Moderate glaucoma (C) (n=12)	Severe glaucoma (D) (n=15)	p-value
wdVD	56.5 ± 2.4^a,b,c^	52.6 ± 4.0^d^	46.9 ± 8.0	43 ± 9.23	<0.001
idVD	57.17 ± 4.13^a,b,c^	51.85 ± 7.15	51.46 ± 4.11	46.22 ± 9.23	<0.001
ppVD	59.92 ± 2.50^a,b,c^	55.90 ± 4.30^d^	49.38 ± 9.77	44.85 ± 11.55	<0.001
Superior hemi (ppVD)	60.19 ± 2.87^a,b,c^	56.75 ± 4.01^d^	50.07 ± 10.32	45.17 ± 11.90	<0.001
Inferior hemi (ppVD)	59.62 ± 2.40^a,b,c^	54.94 ± 5.06^d^	48.64 ± 9.67	44.47 ± 11.29	<0.001

**Figure 1:**
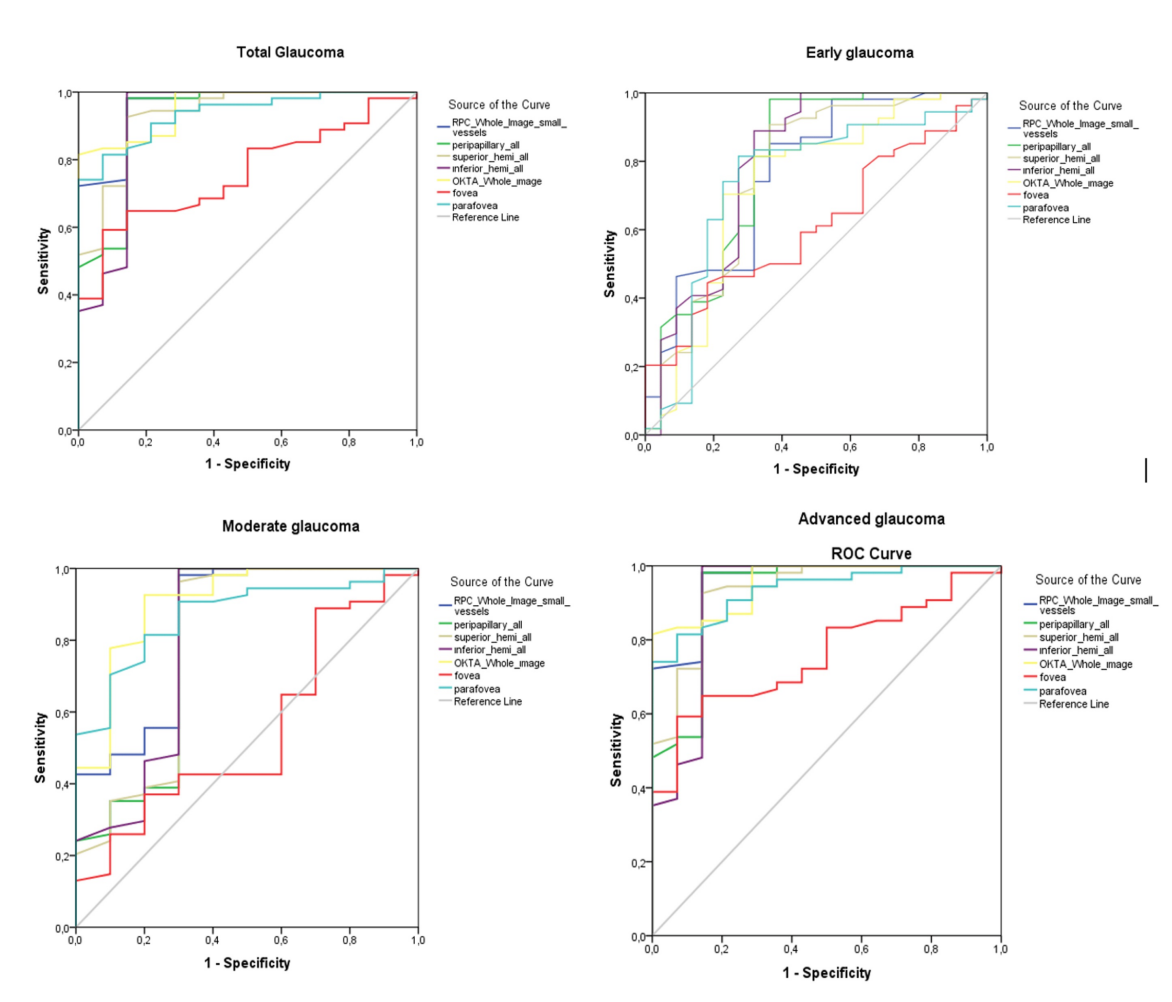
The area under the receiver operating characteristic curves of the OCTA vessel density parameters in the total glaucoma, early glaucoma, moderate glaucoma, and advanced glaucoma groups. RPC: Radial peripapillary capillary; OCTA: Optical coherence tomography angiography; ROC: receiver operating characteristic

The diagnostic performances of the OCTA RPC vessel density parameters and EM vessel density parameters calculated by AUCs between the control and the glaucoma groups are presented in Table 3 and Figure 1.

**Table 3:** Diagnostic performances of OCTA vessel density parameters in differentiating between control and glaucoma groups. Data is expressed as AUC with a 95% confidence interval. wdVD: Whole en face disc vessel density; OCTA: Optical coherence tomography angiography; ppVD: Peripapillary vessel density; EM: En face macular; pafVD: Parafoveal vessel density; fVD: Foveal vessel density; wmVD: Whole macula vessel density; RPC: Radial peripapillary capillary

	Glaucoma (n=51)	Early glaucoma (n=24)	Moderate glaucoma (n=12)	Severe glaucoma (n=15)
OCTA: Vessel density				
RPC				
wdVD	0.859 (0.784-0.934)	0.765 (0.639-0.891)	0.844 (0.692-0.997)	0.959 (0.906-0.999)
ppVD average	0.834 (0.748-0.919)	0.790 (0.658-0.921)	0.799 (0.604-0.994)	0.927 (0.836-0.999)
Superior hemi (ppVD)	0.822 (0.734-0.909)	0.764 (0.628-0.900)	0.794 (0.598-0.989)	0.933 (0.857-0.999)
Inferior hemi (ppVD)	0.834 (0.747-0.921)	0.796 (0.665-0.927)	0.802 (0.608-0.996)	0.917 (0.807-0.999)
EM				
wmVD	0.838 (0.755-0.921)	0.731 (0.592-0.871)	0.906 (0.797-0.999)	0.956 (0.909-0.999)
fVD	0.642 (0.534-0.749)	0.610 (0.479-0.742)	0.547 (0.355-0.739)	0.759 (0.640-0.878)
pafVD average	0.827 (0.741-0.914)	0.739 (0.601-0.877)	0.870 (0.768-0.973)	0.936 (0.878-0.993)

Generally, one of the RPC vessel density parameters, wdVD, had the highest diagnostic performance in all POAG patients (AUC: 0.859). In the moderate glaucoma group, wmVD had the best diagnostic performance (AUC: 0.906). In the severe glaucoma groups, wdVD and wmVD had similar diagnostic performance, AUC: 0.959 and AUC: 0.956, respectively.

In the sectoral ppVD AUCs, we found that the inferior hemi (AUC: 0.796 (0.665-0.927)) and the average ppVD (AUC: 0.790 (0.658-0.921)) had the best AUCs for the detection of early glaucoma. The parameter with the lowest diagnostic performance was the fovea. Although the fovea was not successful in differentiating between control and early/moderate glaucoma, the AUC obtained in differentiating severe glaucoma was 0.759 (95% CI: 0.640-0.878).

The correlation analysis revealed that OCTA RPC and EM vessel density parameters were significantly correlated with the visual field indices MD, corresponding sectoral RNFL, and GCC in different stages of glaucomatous eyes. The correlation between OCTA vessel density parameters and RNFL was more remarkable (Table 4). The most significant correlation was between ppVD and RNFL (0.833, p < 0.001). RNFL and GCC were the other significant parameters correlated with the MD (r = -0.710, p < 0.001 and r = -0.614, p < 0.001, respectively).

**Table 4:** Correlation between OCTA vessel density parameters and other parameters in the glaucomatous eyes. Data is expressed as the Spearman’s rank correlation coefficient (r) with the p-value in the parentheses. OCTA: Optical coherence tomography angiography; RPC: Radial peripapillary capillary; EM: En face macular; wdVD: Whole en face disc vessel density; ppVD: Peripapillary vessel density; wmVD: Whole en face macula vessel density; pafVD: Parafoveal vessel density; MD: Mean deviation; RNFL: Retinal nerve fiber layer; GCC: Ganglion cell complex

	RPC wdVD		RPC ppVD average		EM wmVD		EM pafVD average
MD	0.689 (<0.001)		0.698 (<0.001)		0.656 (<0.001)		0.589 (<0.001)
RNFL	0.792 (<0.001)		0.833 (<0.001)		0.732 (<0.001)		0.606 (<0.001)
GCC	0.674 (<0.001)		0.676 (<0.001)		0.700 (<0.001)		0.572 (<0.001)

## Discussion

In this study, we evaluated the vascular parameters in patients with different stages of POAG using OCTA. Our findings demonstrate significant alterations in both peripapillary and macular regions, with progressive vessel density reduction correlating with structural and functional damage. Measurements of the RPC vessel density parameters, ppVD and wdVD, appear to provide useful diagnostic information and have become the main focus of research in this field [6]. Similarly to our study, ppVD and wdVD are more concordant, and a significant decrease has been found in previous studies [5-10,13]. A study by Kumar et al. also showed a significant decrease in wdVD in the group of pre-perimetric glaucoma [14].

We demonstrated that ppVD and wdVD possess a robust capacity to distinguish between glaucomatous and healthy eyes. Similarly, Yarmohammadi et al. [15] reported a significant diagnostic power of these parameters, with AUCs of 0.94 and 0.83, respectively. In our study, a sectoral analysis of RPC vessel density AUCs for early glaucoma revealed that the inferior hemisphere exhibited the highest AUCs. Numerous studies have similarly identified the inferior hemisphere (AUC = 0.83 ± 0.03) as the most promising region for distinguishing early glaucoma.

In line with our study, pfVD and wmVD also demonstrated a decrease in glaucoma, corresponding with the severity of glaucoma as shown with using the same protocol (SSADA) and 6x6 macular imaging, in a study by Takusagawa et al. [16]. Nonetheless, we observed no significant difference in fVD between the control group and various stages of glaucoma. This could be attributed to the foveal vascular area since its presence occupies a substantial portion of the surface area of fVD.

Rao et al. [17] investigated the diagnostic accuracy of macular circulation and reported a relatively low AUC value of 0.69. A key difference from our study lies in the area calculated in the macular region (3×3 mm in their study vs. 6.6 mm in ours), where their relatively smaller scan area may have overlooked peripheral ganglion cell involvement. Notably, the macular regions most susceptible to glaucoma, particularly the temporal and inferotemporal areas, are primarily situated outside the central 3.3 mm field but within the broader 6.6 mm field [18].

Our analysis revealed a topographic relationship between structural damage, vascular impairment, and functional deficits, highlighting a spatial correlation among regions of vascular disruption, structural changes, and visual field abnormalities. Quantitatively, we identified significant correlations across these parameters, aligning with existing literature and showing consistent correlation and similar AUCs between OCTA measurements, especially ppVD, and RNFL values [19]. However, Yarmohammadi et al. and Khayrallah et al. [5,6] reported a more significant correlation between OCTA parameters and visual field indices rather than RNFL measurements. Several factors may account for this difference. Firstly, it may partly stem from the presence of dysfunctional (pre-apoptotic) (RGCs that exhibit reduced blood flow, lower vessel density, and decreased visual field sensitivity since these cells have not yet atrophied, and reductions in RNFL thickness and rim area may remain undetectable [5]. Furthermore, histological studies have shown [20,21] only moderate agreement between RNFL thinning and RGC loss, indicating that RNFL thinning may not fully represent the functional state of RGCs. Consequently, the stronger correlation between vessel density and visual field damage may imply that vessel density better reflects RGC functionality than structural loss [5].

Notably, our study excludes patients with systemic diseases, thus isolating the vascular changes specific to POAG. This exclusion distinguishes our findings from those that may include confounding vascular impacts of systemic conditions like hypertension or diabetes [6,7,22]. The diagnostic capability of OCTA parameters, particularly ppVD, is evident in early-stage POAG detection. Studies by Liu et al. [10] and Richter et al. [23] reported that peripapillary perfusion parameters outperform macular parameters in diagnostic accuracy, underscoring the critical role of vascular changes in the peripapillary region as a primary marker in glaucoma.

Our study has several advantages; however, it is imperative to recognise its constraints. The number of people in our group was smaller than in some similar studies. Future research could benefit from larger, multi-center studies that allow for more generalizable conclusions. When the OCTA images were taken, the majority of the glaucoma patients were already taking medication to reduce IOP. The impact of IOP-reducing medicine must be considered while interpreting the OCTA parameters. Patients suspected of having glaucoma were not enrolled in our study. The importance of OCTA in glaucoma detection should have been better shown in this case.

## Conclusions

In conclusion, our study highlights the diagnostic value of combining structural and vascular parameters in the assessment of glaucoma. The integration of RNFL, GCC, and OCTA vessel density metrics offers a comprehensive approach to monitoring disease progression. While previous studies have contributed valuable insights, our focus on a homogenous population and advanced imaging techniques provides a clearer understanding of glaucoma’s impact on retinal microcirculation. These findings suggest that OCTA should be used in conjunction with traditional structural assessments of glaucoma.

## References

[1] Quigley HA, Broman AT (2006). The number of people with glaucoma worldwide in 2010 and 2020. Br J Ophthalmol.

[2] Bussel II, Wollstein G, Schuman JS (2014). OCT for glaucoma diagnosis, screening and detection of glaucoma progression. Br J Ophthalmol.

[3] Flammer J (1994). The vascular concept of glaucoma. Surv Ophthalmol.

[4] Grieshaber MC, Flammer J (2005). Blood flow in glaucoma. Curr Opin Ophthalmol.

[5] Yarmohammadi A, Zangwill LM, Diniz-Filho A (2016). Relationship between optical coherence tomography angiography vessel density and severity of visual field loss in glaucoma. Ophthalmology.

[6] Khayrallah O, Mahjoub A, Ben Abdesslam N (2021). Optical coherence tomography angiography vessel density parameters in primary open-angle glaucoma. Ann Med Surg (Lond).

[7] Yarmohammadi A, Zangwill LM, Manalastas PI (2018). Peripapillary and macular vessel density in patients with primary open-angle glaucoma and unilateral visual field loss. Ophthalmology.

[8] Manalastas PI, Zangwill LM, Daga FB (2018). The association between macula and ONH optical coherence tomography angiography (OCT-A) vessel densities in glaucoma, glaucoma suspect, and healthy eyes. J Glaucoma.

[9] Yarmohammadi A, Zangwill LM, Diniz-Filho A (2017). Peripapillary and macular vessel density in patients with glaucoma and single-hemifield visual field defect. Ophthalmology.

[10] Liu L, Jia Y, Takusagawa HL (2015). Optical coherence tomography angiography of the peripapillary retina in glaucoma. JAMA Ophthalmol.

[11] Shoji T, Zangwill LM, Akagi T (2017). Progressive macula vessel density loss in primary open-angle glaucoma: a longitudinal study. Am J Ophthalmol.

[12] Jia Y, Tan O, Tokayer J (2012). Split-spectrum amplitude-decorrelation angiography with optical coherence tomography. Opt Express.

[13] Akil H, Huang AS, Francis BA, Sadda SR, Chopra V (2017). Retinal vessel density from optical coherence tomography angiography to differentiate early glaucoma, pre-perimetric glaucoma and normal eyes. PLoS One.

[14] Kumar RS, Anegondi N, Chandapura RS (2016). Discriminant function of optical coherence tomography angiography to determine disease severity in glaucoma. Invest Ophthalmol Vis Sci.

[15] Yarmohammadi A, Zangwill LM, Diniz-Filho A (2016). Optical coherence tomography angiography vessel density in healthy, glaucoma suspect, and glaucoma eyes. Invest Ophthalmol Vis Sci.

[16] Takusagawa HL, Liu L, Ma KN (2017). Projection-resolved optical coherence tomography angiography of macular retinal circulation in glaucoma. Ophthalmology.

[17] Rao HL, Pradhan ZS, Weinreb RN (2017). A comparison of the diagnostic ability of vessel density and structural measurements of optical coherence tomography in primary open angle glaucoma. PLoS One.

[18] Edlinger FS, Schrems-Hoesl LM, Mardin CY, Laemmer R, Kruse FE, Schrems WA (2018). Structural changes of macular inner retinal layers in early normal-tension and high-tension glaucoma by spectral-domain optical coherence tomography. Graefes Arch Clin Exp Ophthalmol.

[19] Chung JK, Hwang YH, Wi JM, Kim M, Jung JJ (2017). Glaucoma diagnostic ability of the optical coherence tomography angiography vessel density parameters. Curr Eye Res.

[20] Munguba GC, Galeb S, Liu Y (2014). Nerve fiber layer thinning lags retinal ganglion cell density following crush axonopathy. Invest Ophthalmol Vis Sci.

[21] Chauhan BC, Stevens KT, Levesque JM (2012). Longitudinal in vivo imaging of retinal ganglion cells and retinal thickness changes following optic nerve injury in mice. PLoS One.

[22] Triolo G, Rabiolo A, Shemonski ND (2017). Optical coherence tomography angiography macular and peripapillary vessel perfusion density in healthy subjects, glaucoma suspects, and glaucoma patients. Invest Ophthalmol Vis Sci.

[23] Richter GM, Chang R, Situ B (2018). Diagnostic performance of macular versus peripapillary vessel parameters by optical coherence tomography angiography for glaucoma. Transl Vis Sci Technol.

